# Immune activation despite preserved CD4 T cells in perinatally HIV-infected children and adolescents

**DOI:** 10.1371/journal.pone.0190332

**Published:** 2017-12-29

**Authors:** Patricia Alvarez, Mussa Mwamzuka, Fatma Marshed, Adam Kravietz, Tiina Ilmet, Aabid Ahmed, William Borkowsky, Alka Khaitan

**Affiliations:** 1 HIV-1 Molecular Epidemiology Laboratory, Department of Microbiology and Parasitology, Hospital Ramon y Cajal-IRYCIS and CIBERESP, Madrid, Spain; 2 Bomu Hospital, Comprehensive Care Centre, Mombasa, Kenya; 3 Department of Microbiology, New York University School of Medicine, New York, NY, United States of America; 4 Department of Pediatrics, Division of Infectious Diseases and Immunology, New York University School of Medicine, New York, NY, United States of America; George Washington University, UNITED STATES

## Abstract

**Background:**

HIV disease progresses more rapidly in children than adults with mortality rates exceeding 50% by 2 years of age without antiretroviral therapy (ART) in sub-Saharan Africa. Recent World Health Organization (WHO) guidelines recommend universal treatment for all living persons with HIV, yet there is limited supporting evidence in pediatric populations. The objective of this study was to determine whether CD4 cell counts reflect immunological markers associated with disease progression in ART naïve perinatally-infected HIV+ children and adolescents and their response to ART.

**Methods:**

PBMC and plasma samples were collected from 71 HIV negative and 132 HIV+ children (65 ART naïve and 67 on ART) between ages 1–19 years from Mombasa, Kenya. Untreated HIV+ subjects were sub-categorized by high or low CD4 T cell counts. Immune activation markers CD38, HLA-DR and Ki67 were analyzed by flow cytometry. Plasma soluble CD14 (sCD14) was quantified by ELISA.

**Results:**

HIV-infected children and adolescents with preserved CD4 cell counts had depleted CD4 percentages and CD4:CD8 ratios, and high immune activation levels. ART initiation rapidly and persistently reversed T cell activation, but failed to normalize CD4:CD8 ratios and plasma sCD14 levels.

**Conclusions:**

Diminished CD4 percentages and CD4:CD8 ratios along with profound immune activation occur independent of CD4 cell count thresholds in ART naïve HIV+ children and adolescents. Immediate ART initiation, as recommended in the most recent WHO guidelines may protect them from pathologic sequelae associated with persistent inflammation.

## Introduction

In 2016, 2.1 million children under 15 years old were living with HIV, comprising 6% of the total HIV-infected population [1]. Without any therapeutic intervention, 52% of HIV-infected infants in sub-Saharan Africa die before the age of two years [2]. Therefore, an early HIV diagnosis in infants followed by antiretroviral therapy (ART) initiation is essential to reduce HIV-related mortality and long-term morbidity [3]. However, long-term ART entails associated problems such as toxicity, poor adherence, and development of drug resistance.

In recent years, the World Health Organization (WHO) has sequentially modified guidance documents to recommend earlier antiretroviral therapy initiation. The 2013 WHO guidelines for adolescents aged 10–19 years were revised to match those for adults, recommending treatment when CD4 counts are less than 500 cells/mm^3^ [4]. In children ages 5–10 years, treatment initiation was recommended for CD4 counts less than 500 cells/mm^3^ and for all children with WHO clinical stage 3 or 4 disease regardless of CD4 cell counts [4]. ART was recommended for all children less than five years of age, regardless of clinical or immunological status, because of rapid disease progression and high mortality in this population [4]. In 2015, The Strategic Timing of Antiretroviral Therapy (START) clinical trial provided evidence of the positive effect that ART had on the health of HIV-infected adult populations when initiated for CD4 counts above 500 cells/mm^3^ compared to delayed treatment. Early ART resulted in reduced AIDS-related and serious non AIDS-related events, highlighting the benefits of immediate therapy after HIV diagnosis [5]. Considering the results of the START study, in 2016 the WHO revised its guidelines and recommended treatment initiation for all living persons diagnosed with HIV regardless of symptoms or clinical stage [6]. However, there is limited evidence supporting this recommendation for children and adolescents with perinatal HIV infection [5].

Thus far, only two randomized trials have addressed the effect of early ART on infants and children. The Children with HIV Early Antiretroviral Therapy (CHER) trial showed a reduction in infant mortality and HIV progression in asymptomatic perinatally infected infants aged 6 to 12 weeks starting ART immediately compared to those starting ART for CD4 levels below 20% or WHO stage 3 or 4 [3]. Additional studies from the CHER trial showed that early treatment in infants less than 1 year of age preserves immune function, prevents clinical progression and improves immunological and neurodevelopmental outcomes [7, 8]. The Pediatric Randomized Early versus Deferred Initiation in Cambodia and Thailand (PREDICT) clinical trial, which enrolled HIV-positive children aged 1–12 years, showed high AIDS-free survival in both early (CD4 15–24%) and deferred ART (CD4 <15%) groups [9]. While the CHER trial demonstrated the beneficial impact of early ART in HIV-infected infants, the results from PREDICT study detected no difference in mortality between early and deferred ART in children; however the PREDICT study was underpowered to detect a difference in mortality due to low overall mortality and high AIDS-free survival rates in both groups.

Much of the non-AIDS related morbidity in both adults and children is associated with chronic inflammation. Indeed, T cell activation, marked by CD38 and HLA-DR coexpression on CD8 T cells is a prognostic indicator stronger than CD4 T cell counts for disease progression at different stages of HIV infection [10–16]. Another immune activation marker that reflects cell cycling, Ki67, is also associated with HIV-1 disease progression in adults [11, 13, 15], despite prolonged stable CD4 T cell counts after seroconversion [17]. One of the main factors driving excessive T cell activation in patients with chronic HIV infection is the circulation of microbial products translocated from the gut, which also occurs in HIV-infected infants [18–22]. Soluble CD14 (sCD14), released after monocyte activation has been used as a surrogate marker of microbial translocation, and was demonstrated as a predictor of mortality in HIV+ adults on treatment [23, 24].

Because the 2016 WHO revised guideline for treatment regardless of clinical and immunological status lacks supporting evidence in pediatric populations, the recommendations for children and adolescents from ages 1 to 19 years were noted as conditional recommendations with low-quality evidence [25]. We hypothesize that immune activation and other factors associated with HIV disease progression, such as inverted CD4:CD8 ratios, occur despite preserved CD4 T cell levels in HIV-infected children. The primary objective of this study was to evaluate immunological markers associated with HIV disease progression, including CD38, HLA-DR, and sCD14, in ART naïve HIV+ children and adolescents with either high or low CD4 counts. Our secondary objective was to evaluate whether ART initiation restores disrupted CD4:CD8 ratios, sCD14, and T cell activation. In a cohort of 132 HIV+ Kenyan children and adolescents, we found diminished CD4 percentages and CD4:CD8 ratios along with remarkable immune activation that occurred independent of CD4 cell count thresholds, demonstrating immunological evidence in support of the recently released WHO revised guidelines to initiate antiretroviral drugs for all children and adolescents with HIV infection [25].

## Materials and methods

### Participants

Ethical approval for this study was obtained from New York University School of Medicine Institutional Review Board (10–02586) and Kenyatta National Hospital / University of Nairobi Ethics and Research Committee (P283/07/2011). Written informed consent and verbal assent when appropriate was obtained from all participants/parents. Individuals with a recent acute illness, fever, active *Mycobaterium tuberculosis* or malaria infection, or pregnancy within one year were not eligible for study entry. Plasma and peripheral blood mononuclear cells (PBMC) were isolated from peripheral blood by centrifugation and Ficoll-Hypaque (GE Healthcare) density gradient centrifugation then cryopreserved in -80°C and liquid nitrogen respectively. HIV RNA PCR was performed on diluted plasma samples with Roche, COBAS^®^ AmpliPrep/COBAS^®^ TaqMan^®^HIV-1 Test, version 2.0 (limit of detection 110 copies/ml).

### Subjects and categories

We enrolled a total of 71 HIV negative-unexposed children and 132 perinatally-infected HIV+ children between ages 1–19 years from Bomu Hospital in Mombasa, Kenya, between 2011 and 2012. HIV+ children included 65 who had no prior antiretroviral therapy (**ART-**) and 67 on antiretroviral treatment for at least six months (**ART+**). For analyses, participants were divided in two groups according to age: **children** (1 to <10 years old) and **adolescents** (10 to 19 years old). ART- subjects were sub-categorized according to CD4 T cell counts: **ART-CD4**_**hi**_ (>750 cells/mm^3^ for children <5 years; >500 cells/mm^3^ for children ≥5 years) and **ART-CD4**_**lo**_ (≤750 cells/mm^3^ for children <5 years; ≤500cells/mm^3^ for children ≥5 years). These sub-categories were based upon previous WHO recommendations of CD4 thresholds to initiate ART in children. Subject characteristics are shown in Table 1. HIV-, ART-CD4_hi_, ART-CD4_lo_, and ART+ children and adolescents were matched for age and gender.

**Table 1:** Subject characteristics.

	HIV-	ART- CD4_hi_^^^	ART- CD4_lo_^#^	ART+	p
**CHILDREN** (Ages 1-<10 years)					
n	41	32	11	44	
Age (years)*	4 (3–6)	5 (2–8)	3 (2–9)	4 (3–7)	NS^a^
Female (n)	24 (59%)	17 (53%)	2 (18%)	21 (548%)	NS^b^
CD4 cells/mm3*	1303 (900–1660)	1225 (775–1504)	383 (252–537)	1344 (967–1812)	p<0.0001^a^
**ADOLESCENTS** (Ages 10–19 years)					
n	31	11	11	23	
Age (years)*	12 (11–16)	14 (12–15)	13 (12–15)	13 (12–16)	NS^a^
Female	13 (43.3%)	7 (64%)	6 (54.5%)	11 (48%)	NS^b^
CD4 cells/mm3*	908 (674–1146)	645 (514–755)	285 (71–396)	765 (492–1116)	p<0.0001^a^

^ CD4 >750 cells/mm3 for children <5 years; CD4 >500 cells/mm3 for children ≥5 years

# CD4 ≤750 cells/mm3 for children <5 years;CD4 ≤500cells/mm3 for children ≥5 years

*Median values with upper and lower quartile range

(a) Kruskal-Wallis test

(b) Chi-squared test

### Flow cytometric studies

PBMCs were stained with fixable viability dye (eBioscence) in PBS for 30 minutes then stained with fluorescent-conjugated antibodies at 4°C for 30 minutes in PBS buffer containing 2% FCS and 0.1% sodium azide. For intracellular staining, cells were fixed and permeabilized with eBioscience kit per manufacturer's instructions. Stained cells were analyzed using LSRII flow cytometer (BD Bioscience) and FlowJo software (Tree Star). The following anti-human antibodies were used: CD3, CD4, CD8, CD38, CD45RO, HLA-DR and Ki67 (Biolegend). All populations were gated on live cells and are reported as percent of CD4 or CD8 T cell parent populations.

### Plasma sCD14

Plasma levels of sCD14 were quantified by ELISA assay using Human CD14 Duoset kit (R&D Systems) per manufacturer’s instructions. Plasma was diluted 1500 fold and each test performed in duplicate. Results reported are the average of duplicate results.

### Statistics

All statistical analyses were performed using GraphPad Prism software. Comparisons between participant categories were computed with the two-sided Mann-Whitney test or the Kruskal-Wallis test corrected for multiple comparisons by controlling the False Discovery Rate with the Benjamini, Krieger, and Yekutieli test. Data from multiple time points were evaluated with the Wilcoxon matched-pairs signed rank test. For linear regression analysis, log CD38+HLADR+ CD8 T cell frequencies were treated as the dependent variable and absolute CD4 cell count as the independent variable. Threshold of significance for all tests was 0.05.

## Results

### HIV disease progression in ART-CD4_hi_ children and adolescents

**Fig 1:**
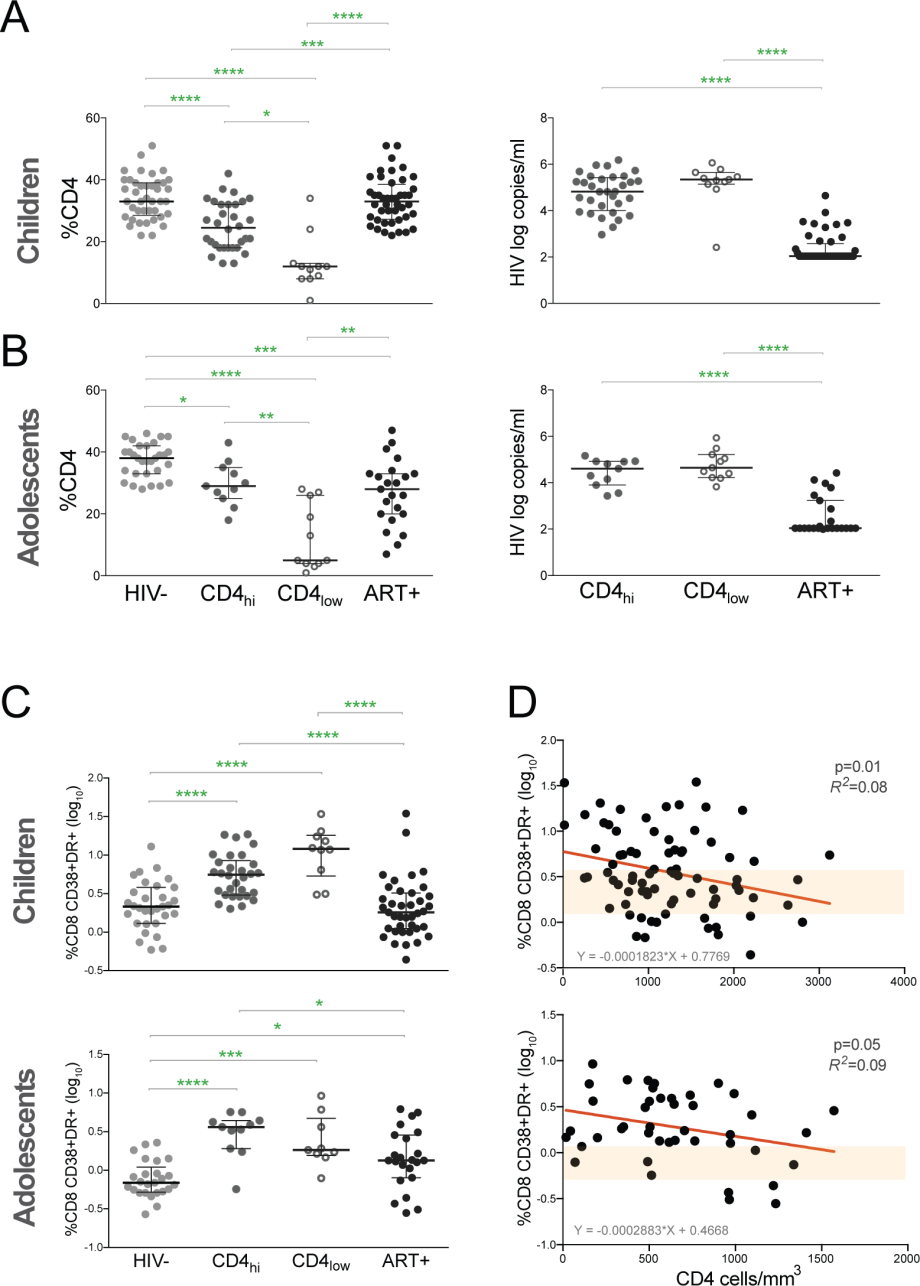
HIV disease progression in ART-CD4_hi_ children and adolescents. Comparison of the **(A)** CD4 percent and **(B)** HIV viral load in HIV-, ART-CD4_hi_, ART- and ART+ children and adolescents. (C) Comparison of the %CD38+DR+ CD8 T cells in HIV-, ART-CD4_hi_, ART-CD4_lo_ and ART+ children and adolescents. Bars represent median values with IQRs. P values were calculated using the Kruskal-Wallis test corrected for multiple comparisons by controlling the false discovery rate with the Benjamini, Krieger, and Yekutieli test. **** p<0.0001; *** p<0.001; ** p<0.01; * p<0.05. (D) The percent of CD38+DR+ CD8 T cells vs. CD4 cell count in total lymphocytes in children and adolescents. P and *R*^*2*^ values are shown for a linear regression model. Shaded bar represents interquartile range of CD38+HLA-DR+ CD8 T cell frequencies in HIV-uninfected children.

As absolute CD4 T cell counts vary with age, we assessed the CD4 percentage in each subject group [26]. In children, ART-CD4_hi_ had lower CD4 percentages than HIV- and ART+ (p<0.0001; Fig 1A), but higher levels compared to ART-CD4_lo_ children (p = 0.02; Fig 1A). In adolescents, ART-CD4_hi_ also had decreased CD4 percentages compared to HIV- (p = 0.01; Fig 1B) but did not differ from ART+. ART-CD4_hi_ children had median HIV viral load of 4.8 log copies/ml while ART-CD4_lo_ children had median viral load of 5.3 log copies/ml (Fig 1A). ART-CD4_hi_ and ART-CD4_lo_ adolescents had median HIV viral loads of 4.6 log copies/ml (Fig 1B). Both ART+ children and adolescents had median HIV copies of 2 log copies/mL, which was the limit of detection (Fig 1A and 1B). For both children and adolescents, there was no significant difference in HIV viral load between ART-CD4_hi_ and ART-CD4_lo_ groups.

Because coexpression levels of CD38 and HLA-DR on CD8 T cells predict HIV disease progression, we compared these markers between subject categories (gating strategy shown in S1 Fig). ART-CD4_hi_ children had higher CD38^+^HLA-DR^+^ frequencies in CD8 T cells compared to HIV- and ART+ (p<0.0001) and similar frequencies compared to ART-CD4_lo_ (Fig 1C). CD38^+^HLA-DR^+^ CD8 T cell levels were similar between ART+ and HIV- children. In adolescents, ART-CD4_hi_ had increased CD8+ CD38^+^HLA-DR^+^ frequencies compared to HIV- (p<0.0001) and ART+ (p = 0.02; Fig 1C) and no significant difference compared to ART-CD4_lo_ (Fig 1C). ART+ adolescents also had higher levels of CD38+HLA-DR+ CD8 T cells compared to HIV- adolescents (p-0.02; Fig 1C). To determine whether CD4 counts predict immune activation in CD8 T cells we performed a linear regression analysis. In children, CD4 cell counts predicted the proportion of CD38+HLADR+ CD8 T cells (p = 0.01, *R*^*2*^ = 0.08; Fig 1D). Based on the regression algorithm, CD4 cell counts of 2438 and 1075 would predict the median and upper quartile frequencies of CD38+HLA-DR+ CD8 T cells in healthy children. For adolescents, the regression model trended towards significance (p = 0.05, *R*^*2*^ = 0.09; Fig 1D), and CD4 cell counts of 1475 would correspond to the upper quartile of CD38+HLA-DR+ CD8 T cell frequencies in HIV- adolescents. These algorithms suggest that a high CD4 threshold for treatment initiation would be necessary to prevent CD8 T cell activation in pediatric populations.

### ART-CD4_hi_ children and adolescents have significant CD4 T cell activation

**Fig 2:**
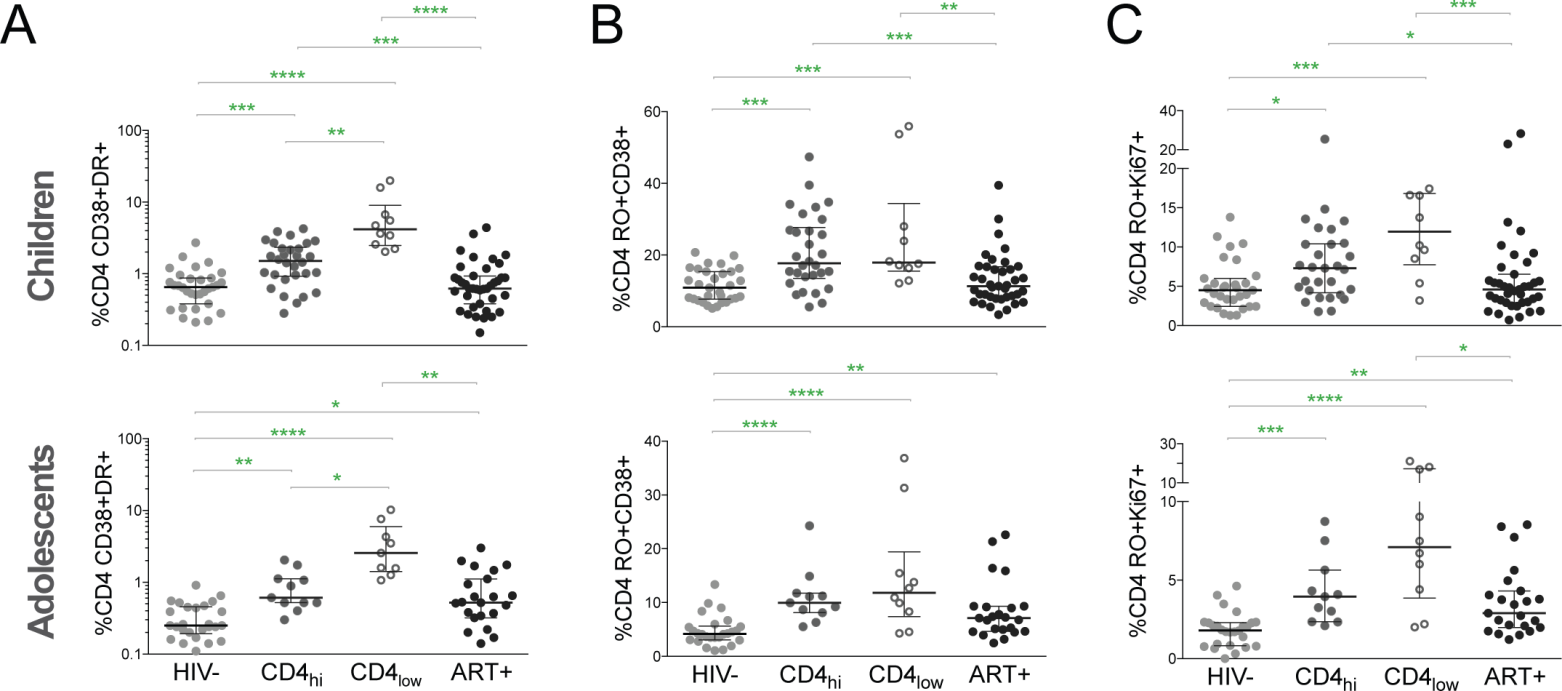
Significant CD4 T cell activation in ART-CD4_hi_ children and adolescents. (A) Comparison of frequencies of the CD38+HLA-DR+ CD4 T cells in HIV-, ART-CD4_hi_, ART-CD4_lo_ and ART+ children and adolescents. Percentages of (B) CD38+ and (C) Ki67+ cells within in memory CD4 T cells in HIV-, ART-CD4_hi_, ART-CD4_lo_ and ART+ children and adolescents. To identify memory populations, CD4 T cell were first gated on CD45RO+ CD4 T cells. Bars represent median values with IQRs. P values were calculated using the Kruskal-Wallis test corrected for multiple comparisons by controlling the false discovery rate with the Benjamini, Krieger, and Yekutieli test. **** p<0.0001; *** p<0.001; ** p<0.01; * p<0.05.

**Fig 3:**
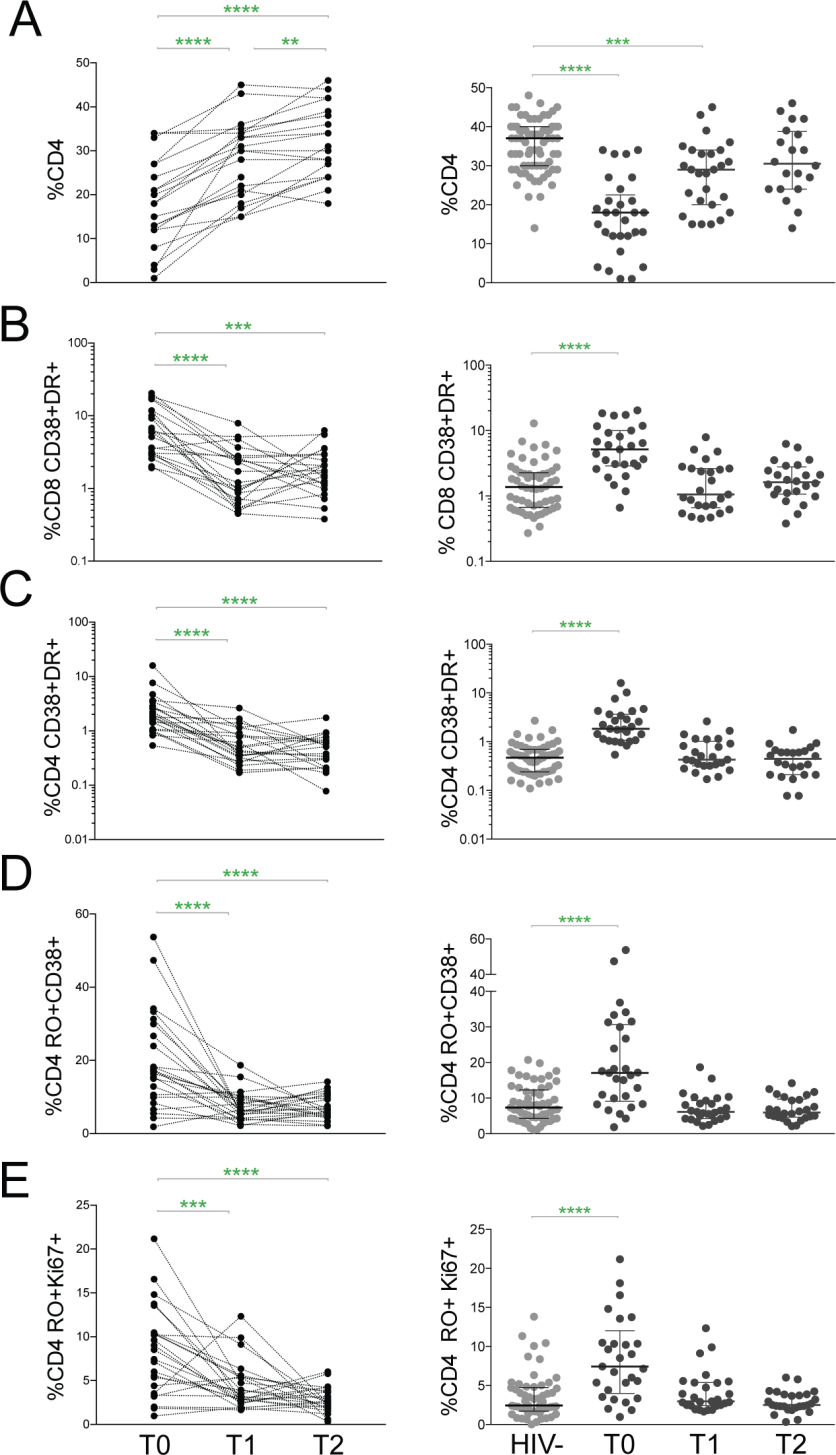
Antiretroviral therapy lowers immune activation rapidly and persistently. Comparisons of **(A)** the CD4 percentages and **(B-E)** frequencies of the following IA markers in paired longitudinal samples pre-ART (T0), 5–7 months post-ART (T1), and 10–16 months post-ART (T2): CD38+HLA-DR+ **(B)** CD8 and **(C)** CD4 T cells and **(D)** CD38+ **(E)** and Ki67+ memory CD4 T cells. Right graphs show comparison between IA markers in HIV- and the prospective cohort pre- and post-ART. Bars represent median values with IQRs. P values were calculated using the paired Wilcoxon matched-pairs signed rank test (left graphs) and the Kruskal-Wallis test corrected for multiple comparisons by controlling the false discovery rate with the Benjamini, Krieger, and Yekutieli test (right graphs). **** p<0.0001; *** p<0.001; ** p<0.01; * p<0.05.

We next analyzed expression of these activation markers, HLA-DR and CD38, on CD4 T cells (S1 Fig), which also correlate with HIV disease progression [15]. ART-CD4_hi_ children had higher CD38^+^HLA-DR^+^ frequencies in CD4 T cells compared to HIV- (p = 0.0003) and ART+ (p = 0.0005), but lower frequencies than ART-CD4_lo_ children (p = 0.009; Fig 2A). There was no significant difference in CD38^+^HLA-DR^+^ CD4 T cell levels between HIV- and ART+ children. In adolescents, ART-CD4_hi_ had increased CD4+ CD38^+^HLA-DR^+^ frequencies compared to HIV- (p = 0.002), lower frequencies than ART-CD4_lo_ (p = 0.047) and similar frequencies to ART+ adolescents (Fig 2A). Because CD38 and Ki67 mark immune activation only in the memory CD4 T cell population, we determined their expression levels within CD45RO+ (memory) CD4 T cells (S1 Fig). In children, CD38^+^ memory CD4 T cell frequencies were similar between ART-CD4_hi_ and ART-CD4_lo_ (Fig 2B). However, ART-CD4_hi_ had higher CD4^+^CD45RO^+^CD38^+^ levels compared to HIV- (p = 0.0001; Fig 2B) and ART+ children (p = 0.0008; Fig 3B). ART-CD4_hi_ children also had decreased CD4^+^CD45RO^+^Ki67^+^ frequencies compared to HIV- (p = 0.01) and ART+ (p = 0.02) but frequencies similar to ART-CD4_lo_ children (Fig 2C). In adolescents, there was also no significant difference between CD38 and Ki67 expression in memory CD4 T cells in ART-CD4_hi_ and ART-CD4_lo_ subjects (Fig 2B and 2C). ART-CD4_hi_ had lower expression of both activation markers on memory CD4 T cells compared to HIV- (CD38+: p<0.0001; Ki67: p = 0.0003) but similar expression compared to ART+ adolescents (Fig 2B and 2C). Thus HIV+ children and adolescents with high CD4 T cells had significant CD4 T cell activation indicated by multiple markers.

### Antiretroviral therapy lowers immune activation levels in HIV+ children and adolescents rapidly and persistently

With our findings of significant immune activation in ART- subjects, we sought to determine whether antiretroviral therapy reverses inflammation in children and adolescents. A portion of subjects enrolled in our study initiated ART according to guidelines from the Kenya Ministry of Health. In these subjects, we evaluated immunologic responses before receiving antiretroviral therapy (T0), 5–7 months post-ART (T1) and 10–16 months post-ART (T2). Because data was similar between children and adolescents for the prospective analysis, we combined all ages; subject characteristics of this prospective cohort are shown in S1 Table. A rapid virologic response was achieved with HIV viral loads decreased by T1 (p<0.0001) and suppression maintained at T2 (S2 Fig). These patients also had increases in CD4 percentages by T1 (p<0.0001), which continued rising through T2 (p = 0.003) at which point they were similar to levels in HIV- children (Fig 3A). Remarkably, every marker of immune activation decreased by T1 (Fig 3B–3E). CD38+HLA-DR+ CD8 and CD4 T cell frequencies dramatically reduced by T1 (p<0.0001) and remained low at T2. Similarly, CD38+ and Ki67+ memory CD4 T cells were significantly lower at T1 (CD38: p<0.0001; Ki67 p = 0.0002) and persisted at T2 (Fig 3C and 3D). ART-CD4_hi_ and ART-CD4_lo_ exhibited similar immunologic recovery with no difference in the fold change of each activation marker between T0 and T2 (S3 Fig). More importantly, each measure of immune activation that was significantly higher than HIV- children before ART (p<0.0001) normalized to levels found in HIV- children by T1 (Fig 3B–3E, right). Thus, antiretroviral therapy rapidly and persistently reversed evidence of HIV disease progression as measured by immune activation markers on both CD8 and CD4 T cells.

### ART-CD4_hi_ children and adolescents exhibit markers of HIV disease progression associated with mortality

**Fig 4:**
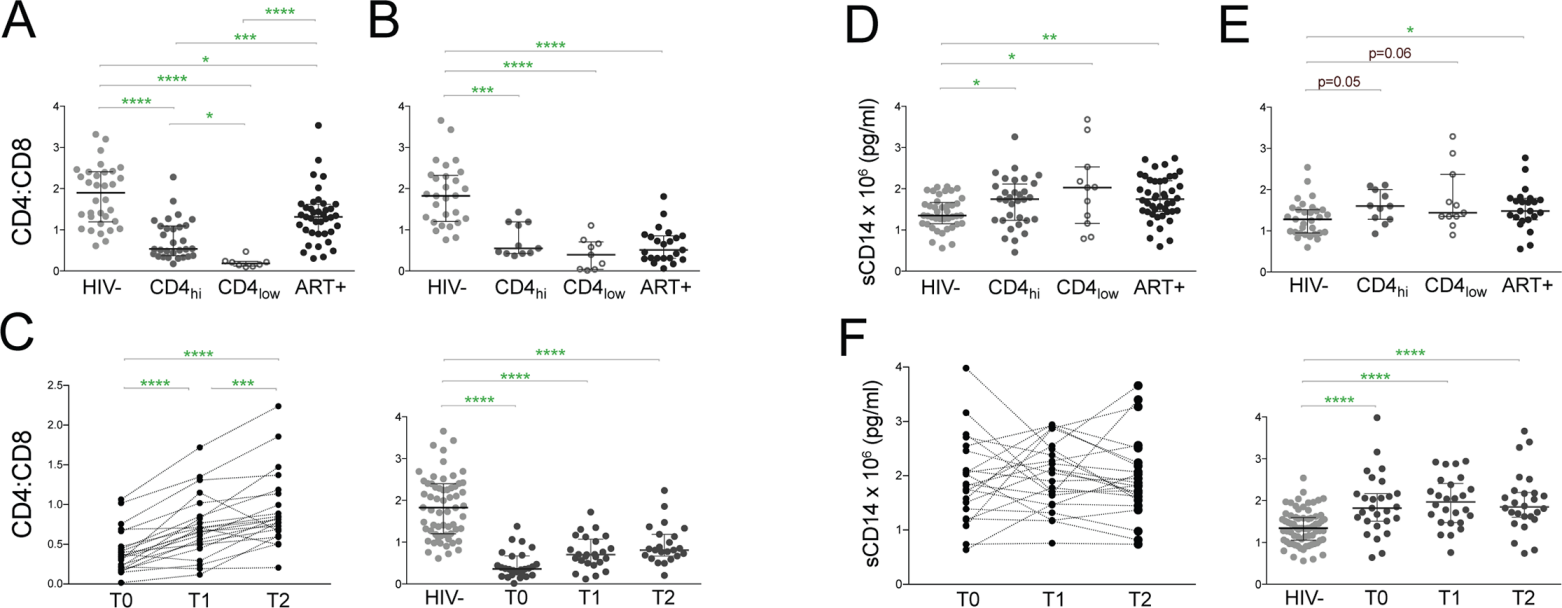
ART-CD4_hi_ children and adolescents exhibit markers of HIV progression associated with mortality. Comparison of the CD4:CD8 ratio in HIV-, ART-CD4_hi_, ART-CD4_lo_ and ART+ **(A)** children and **(B)** adolescents. **(C)** CD4:CD8 ratios are shown in subjects before antiretroviral treatment (T0), 5–7 months post-ART (T1), and 10–16 months post-ART (T2) and in comparison to HIV- children on the right. Plasma sCD14 levels in HIV-, ART-CD4_hi_, ART-CD4_lo_ and ART+ **(D)** children and **(E)** adolescents and **(F)** pre-and post-ART. Bars represent median values with IQRs. P values were calculated using the Kruskal-Wallis test corrected for multiple comparisons by controlling the false discovery rate with the Benjamini, Krieger, and Yekutieli test and the Wilcoxon matched-pairs signed rank test (C and F). **** p<0.0001; *** p<0.001; ** p<0.01; * p<0.05.

During HIV infection, CD8 T cells rise while CD4 cell counts fall, leading to an overall decrease of the CD4:CD8 ratio [27]. Inversion of this CD4:CD8 ratio to less than one has been linked to HIV mortality in studies of adults [27]. In our cohort, HIV+ subjects had decreased CD4:CD8 ratios compared to HIV- subjects, regardless of age group, treatment status or CD4 cell counts (Fig 4A and 4B). ART-CD4_hi_ children had lower CD4:CD8 ratios than HIV- (p<0.0001) and ART+ children (p = 0.0007), but higher ratios than ART-CD4_lo_ children (p = 0.02; Fig 4A). In addition, ART+ children had lower CD4:CD8 ratios than HIV- children (p = 0.02; Fig 4A), despite having similar CD4 T cells percentages (Fig 1A). In the adolescent group, ART-CD4_hi_ had decreased CD4:CD8 ratios compared to HIV- (p = 0.0006) but ratios similar to ART+ adolescents (Fig 4B). ART+ adolescents had significantly lower CD4:CD8 ratios than HIV- adolescents (p<0.0001; Fig 4B). In the prospective cohort, CD4:CD8 ratios increased between T0 and T1 (p<0.0001) and again between T1 to T2 (p = 0.0008; Fig 4C). However, CD4:CD8 ratios remained lower than HIV- subjects at T2 (p<0.0001; Fig 4C).

Plasma sCD14 levels, which reflect monocyte activation after gut microbial translocation, also predict mortality in HIV-infected adults [28]. In our pediatric cohort, ART+ children and adolescents had elevated sCD14 levels compared to HIV- subjects (children: p = 0.002; adolescents p = 0.03; Fig 4D and 4E). ART-CD4_hi_ children had sCD14 levels higher than HIV- (p = 0.03) and similar to ART-CD4_lo_ and ART+ (Fig 4D). sCD14 levels in ART-CD4_hi_ and ART-CD4_lo_ adolescents trended towards higher levels compared to HIV- (p = 0.05 and p = 0.06; Fig 4E). Plasma sCD14 levels in the prospective cohort did not alter within one year of ART initiation and remained elevated compared to HIV- subjects (p<0.0001; Fig 4F).

## Discussion

We demonstrated ART naïve children and adolescents who maintain relatively high CD4 cell counts nonetheless have diminished CD4 percentages, active viral replication, and significant immune activation compared to healthy HIV negative controls. Moreover, these children have inverted CD4:CD8 ratios and elevated sCD14 plasma levels, associated with mortality in HIV infected adults. ART initiation rapidly and persistently reverses systemic immune activation associated with disease progression, but not CD4:CD8 ratios and sCD14 levels that have been linked to mortality [23, 29]. Overall these findings highlight marked inflammation in HIV+ children and adolescents despite preserved CD4 T cells, which has been linked to poor long-term outcomes including impaired neurodevelopment, cardiovascular and metabolic disease, and HIV disease progression [30–32] and potential for normalization with treatment.

In this study we demonstrate that ART-CD4_hi_ HIV+ children and adolescents exhibit substantial immune activation marked by elevated CD38^+^HLA-DR^+^ CD4 and CD8 T cells and increased CD38+ and Ki67+ memory CD4 T cells, despite preserved CD4 T cells. The consequences of chronic immune activation in ART-treated, HIV-infected adults include diseases associated with ageing, such as cardiovascular disease, cerebrovascular accidents, and cancers [33–35], while children exhibit biological aging and immunological senescence [36]. ART-CD4_hi_ and ART-CD4_lo_ children and adolescents had similar proportions of activated CD8 T cells, suggesting CD4 cell counts may not reflect immunologic health. Previous pediatric studies have demonstrated significant associations between CD8 T-cell activation and CD4 percentages and absolute CD4+ cell counts but not with viral load [37–39]. In our study, although CD4 cell counts predicted CD38^+^HLA-DR^+^ CD8 T cell frequencies, the regression models demonstrate CD8 T cell activation begins at a CD4 cell counts over 1000, far above former thresholds for ART initiation. HIV+ children and adolescents also had higher plasma sCD14, providing evidence of gut mucosal disruption and microbial translocation. These results align with prior studies that have shown untreated HIV-infected children have increased levels of sCD14 when compared with healthy controls, the highest levels being observed in the most severely immune-compromised children [19–22, 40].

In our prospective cohort, benefits associated with ART initiation occurred rapidly and were maintained over time. HIV viremia lowered within ~6 months of treatment while CD4% increased by that time and continued rising over the first year after ART initiation. However, CD4 percentages failed to normalize and remained lower than HIV negative controls at one year. Similarly, CD4:CD8 ratios in this cohort continued to increase over the first year of antiretroviral treatment, but did not reach levels of the control population. Impressively, immune activation levels normalized within ~6 months of treatment. Our results are in agreement with previous studies in children that demonstrate ART reduces levels of immune activation [22, 37, 41, 42]. However, in these studies, ART did not normalize immune activation levels. In our cross-sectional analysis, ART+ adolescents, but not children, had elevated frequencies of each immune activation marker compared to HIV- controls. Normalization of these markers in our prospective cohort may be related to a younger age at antiretroviral initiation. Alternatively, our ART- cohort may be a naturally selected subset of children with host genetic factors such as protective HLA alleles, which conferred slow HIV disease progression. This cohort may also have immune protective mechanisms that allowed survival and subsequent rapid decrease in immune activation with antiretroviral therapy. A recent study described pediatric nonprogressors as HIV+ children over 5 years old who maintain CD4 cell counts above 750 cells/mm^3^. These nonprogressors manifested immunologic features similar to nonhuman primate hosts with natural SIV infection [43]. A combination of protective immune and genetic factors likely allowed our ART- cohort to survive in the absence of treatment for several years in a region where mortality of untreated HIV infection exceeds 50% by 2 years of age.

While immune activation markers and CD4 percentages normalized in the prospective cohort, sCD14 and CD4:CD8 ratios failed to reach levels similar to healthy HIV negative subjects. CD4:CD8 ratios increased after ART initiation at both T1 and T2, but remained inverted after one year of treatment and in ART+ subjects. ART also failed to reduce plasma sCD14 levels. Adult studies suggest ART must be started within 12 months of seroconversion to restore immunologic health [44] and early ART initiation might contribute to more rapid and robust CD4:CD8 ratio normalization [29]. In children, one report demonstrated that starting ART before 5 years of age results in stronger CD4 T cell gains but not normalization within 24 months of treatment [34]. A statistical model developed to estimate long-term CD4 recovery, demonstrated that ART should be initiated when CD4-for-age is high, even if there is a low risk of immediate disease progression to maximize CD4 reconstitution in children older than 10 years [45]. Further studies have shown that children with low CD4 counts experience a faster recovery at the beginning, yet the long-term CD4 values remain lower than normal [45, 46]. However in our prospective cohort, with a median age of 8 years, there was no difference in immunologic recovery between ART-CD4_hi_ and ART-CD4_lo_.

The benefits of early ART go beyond the immune recovery. A study conducted in Kenya showed that children who started ART before the age of 3 years were more likely to recover a normal weight [47]. Another study shows better development of the neurological system in children who received early ART [8]. A crucial mediator of these morbidities of delayed growth and neurodevelopment is immune activation, which we demonstrate in untreated HIV+ children and adolescents despite preserved CD4 cell counts. Therefore, early ART initiation may protect them from pathologic sequelae associated with persistent inflammation including impaired growth and neurocognitive development, early cardiovascular disease, metabolic complications, and premature death [30–32].

In conclusion, children and adolescents with preserved CD4 T cells nonetheless have poor immunologic health with persistent inflammation, manifested by low CD4 percentages and CD4:CD8 ratios and significantly elevated immune activation markers. Importantly antiretroviral therapy alleviates systemic immune activation and improves %CD4 and CD4:CD8 ratios, associated with HIV disease progression. Overall CD4 cell counts fail to reflect profound immunologic perturbations in perinatally-infected HIV+ children and adolescents. In the absence of a clear CD4 threshold at which immune disruptions begins, we advocate for ART in all children and adolescents with HIV infection regardless of their CD4 T cell counts or clinical status. These data provide immunological evidence in support of the recent WHO recommendation of treating all children and adolescents living with HIV.

## Supporting information

S1 Fig Flow Cytometry gating strategy for immune activation markers. Representative flow plots gated on CD3+ T cells from a HIV negative child are shown. Cells were gated on CD8+ or CD4+ T cells then for coexpression of CD38 and HLA-DR. Within CD4+ T cells, CD38 and Ki67 were quadrant gated with CD45RO and reported as the positive fraction within total CD45RO+ (memory) CD4 T cells.
(TIF)

S2 Fig Viral suppression after ART in the prospective cohort. Comparison of the viral load in in the prospective cohort before antiretroviral treatment (T0) and 5–7 (T1) and 10–16 (T2 months after treatment. P values calculated with the Wilcoxon matched-pairs signed rank test. **** *p* < 0.0001.
(TIF)

S3 Fig Immunologic changes pre- and post-ART in ART-CD4_hi_ and ART-CD4_low_ subjects. (A-E) The percent change in CD4 T cell levels and activated T cell frequencies after 10–16 months of antiretroviral treatment in ART- CD4_hi_ and CD4_low_ subjects who initiated treatment. Comparisons of the percent change in CD4:CD8 ratios (F) and plasma sCD14 levels (G) in ART- CD4_hi_ and CD4_low_ subjects who initiated treatment. P values were calculated with the Mann Whitney U test with threshold of significance less than 0.05. NS = not significant.
(TIF)

S1 Table Prospective subjects. Demographic and clinical information for HIV-infected subjects included in the prospective analyses before and after antiretroviral treatment.
(PDF)
