# Supplementary figures and images for: Immune activation despite preserved CD4 T cells in perinatally HIV-infected children and adolescents
Source: PLoS One. 2017 Dec 29;12(12):e0190332. doi: 10.1371/journal.pone.0190332 (PMC5747457; doi:10.1371/journal.pone.0190332)

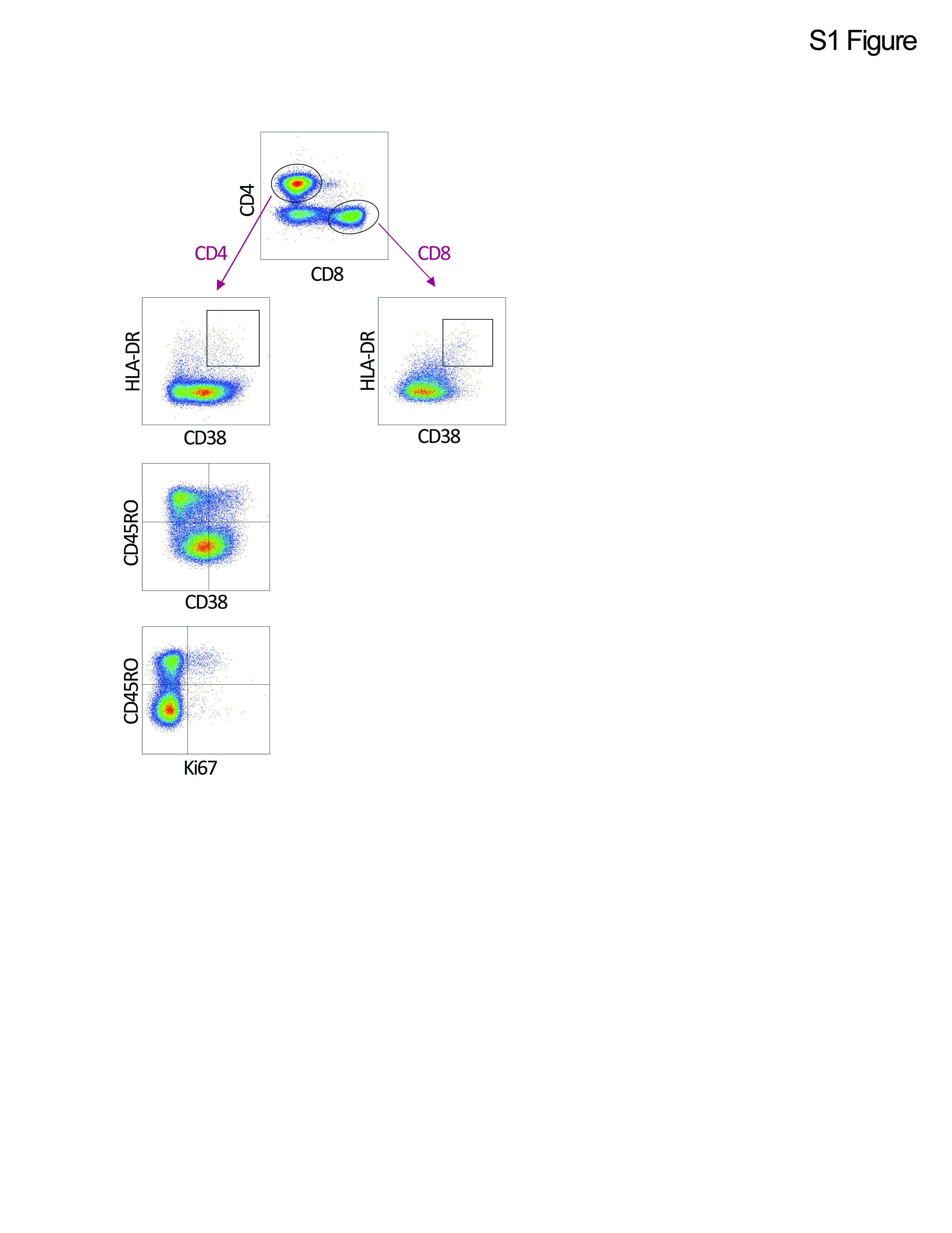

Supplement: S1 Fig — Representative flow plots gated on CD3+ T cells from a HIV negative child are shown. Cells were gated on CD8+ or CD4+ T cells then for coexpression of CD38 and HLA-DR. Within CD4+ T cells, CD38 and Ki67 were quadrant gated with CD45RO and reported as the positive fraction within total CD45RO+ (memory) CD4 T cells. (TIF) [file pone.0190332.s001.tif]

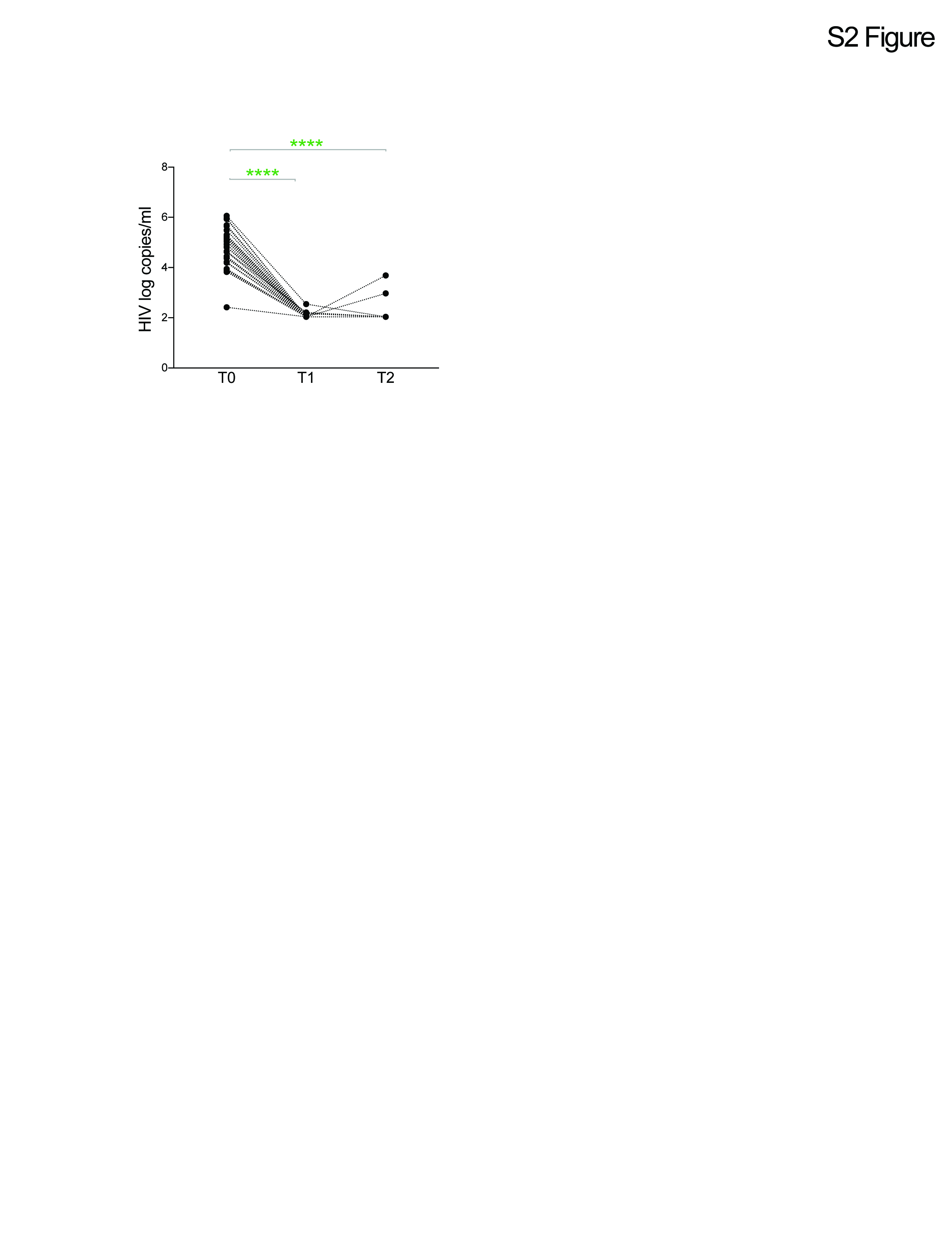

Supplement: S2 Fig — Comparison of the viral load in in the prospective cohort before antiretroviral treatment (T0) and 5–7 (T1) and 10–16 (T2 months after treatment. P values calculated with the Wilcoxon matched-pairs signed rank test. **** p < 0.0001. (TIF) [file pone.0190332.s002.tif]

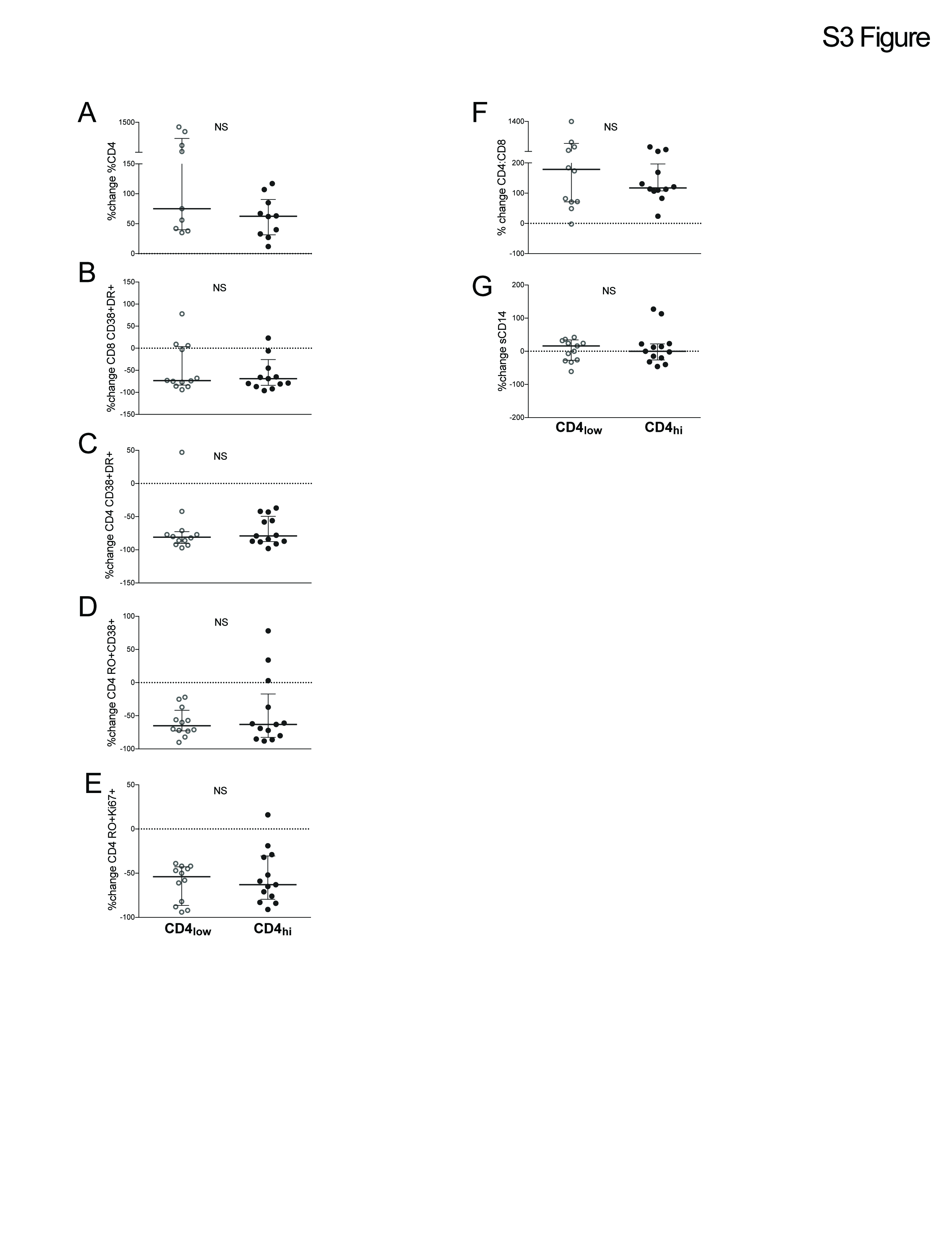

Supplement: S3 Fig — (A-E) The percent change in CD4 T cell levels and activated T cell frequencies after 10–16 months of antiretroviral treatment in ART- CD4hi and CD4low subjects who initiated treatment. Comparisons of the percent change in CD4:CD8 ratios (F) and plasma sCD14 levels (G) in ART- CD4hi and CD4low subjects who initiated treatment. P values were calculated with the Mann Whitney U test with threshold of significance less than 0.05. NS = not significant. (TIF) [file pone.0190332.s003.tif]
