# Supplementary material for: Immune activation despite preserved CD4 T cells in perinatally HIV-infected children and adolescents
Source: PLoS One. 2017 Dec 29;12(12):e0190332. doi: 10.1371/journal.pone.0190332 (PMC5747457; doi:10.1371/journal.pone.0190332)
Supplement: S1 Table — Demographic and clinical information for HIV-infected subjects included in the prospective analyses before and after antiretroviral treatment. (PDF) [file pone.0190332.s004.pdf]

Table S1: Prospective Subjects

PID	Sex	Age (years)	CD4 (cells/mm3)	log HIV copies/mL
239	M	1.0	672	6.06
127	M	1.3	1674	5.96
222	M	1.3	1572	5.69
16	F	1.5	1952	3.91
213	M	1.6	797	3.94
207	M	2.0	537	5.14
215	F	2.0	2104	4.82
24	M	2.5	1385	4.82
201	M	2.8	1240	6.18
225	M	3.4	437	4.93
141	M	5.6	591	3.86
145	M	6.0	524	5.05
94	M	7.4	991	4.45
88	F	7.7	625	5.49
167	F	8.1	545	5.21
169	M	8.5	383	5.30
72	M	8.7	252	5.25
146	F	9.0	648	4.62
157	M	9.1	286	5.65
15	M	9.9	17	2.42
184	M	11.6	285	4.22
142	F	11.8	334	3.83
203	F	12.0	18	4.92
202	F	12.7	396	4.20
209	F	13.7	352	4.40
205	F	14.0	71	5.04
224	M	14.6	175	4.65
180	F	14.9	328	4.37
214	M	14.9	173	5.94
**median (IQR)**	38% F	8 (2-12)	524 (286-594)	4.92 (4.3-5.4)
